# The complete chloroplast genome sequence of *Fagraea fragrans*

**DOI:** 10.1080/23802359.2020.1714504

**Published:** 2020-01-20

**Authors:** Jinfeng Zhang, Yunqing Li, Yi Wang

**Affiliations:** Laboratory of Forest Plant Cultivation and Utilization, Yunnan Academy of Forestry, Kunming, People’s Republic of China

**Keywords:** *Fagraea fragrans*, chloroplast, Illumina sequencing, phylogenetic analysis

## Abstract

The first complete chloroplast genome (cpDNA) sequence of *Fagraea fragrans* was determined from Illumina HiSeq pair-end sequencing data in this study. The cpDNA is 154,416 bp in length, contains a large single copy region (LSC) of 84,405 bp and a small single copy region (SSC) of 18,285 bp, which were separated by a pair of inverted repeats (IR) regions of 25,863 bp. The genome contains 130 genes, including 85 protein-coding genes, 8 ribosomal RNA genes, and 37 transfer RNA genes. The overall GC content of the whole genome is 38.0%, and the corresponding values of the LSC, SSC, and IR regions are 36.0%, 31.8%, and 43.4%, respectively. Further phylogenomic analysis showed that *F. fragrans* in a unique clade in Gentianaceae family.

*Fagraea fragrans* Roxb. belongs to the family of Gentianaceae (Wong and Sugumaran 2012). It is widely distributed throughout Burma to Indo-Malaysia and Thailand. *F. fragrans* is used as traditional medicine in Southeast Asia (Pripdeevech and Saansoomchai 2013). The bark is used as a blood tonic and to treat vesicles. The heartwood is used to treat flatulence, fever, pain at joint, asthma. The leaf extract of *F. fragrans* showed anti-inflammatory (Jonville et al. 2010), antiplasmodial activity (Nguyen-Pouplin et al. 2007), anti-HSV-1 activity, antibacterial and antioxidant activities (Jonville et al. 2008). Therefore, *F. fragrans* has huge medicinal value. However, there have been no genomic studies on *F. fragrans*.

Herein, we reported and characterized the complete *F. fragrans* plastid genome. The GenBank accession number is MN823695. One *F. fragrans* individual (specimen number: 201907027) was collected from Puwen, Yunnan Province of China (23°31′29″N, 101°37′11″E). The specimen is stored at Yunnan Academy of Forestry Herbarium, Kunming, China and the accession number is ZJFEP116. DNA was extracted from its fresh leaves using DNA Plantzol Reagent (Invitrogen, Carlsbad, CA, USA).

Paired-end reads were sequenced by using Illumina HiSeq system (Illumina, San Diego, CA). In total, about 25.1 million high-quality clean reads were generated with adaptors trimmed. Aligning, assembly, and annotation were conducted by CLC de novo assembler (CLC Bio, Aarhus, Denmark), BLAST, GeSeq (Tillich et al. 2017), and GENEIOUS v 11.0.5 (Biomatters Ltd, Auckland, New Zealand). To confirm the phylogenetic position of *F. fragrans*, other seven species of Gentianaceae family from NCBI were aligned using MAFFT v.7 (Katoh and Standley 2013). The Auto algorithm in the MAFFT alignment software was used to align the ten complete genome sequences and the G-INS-i algorithm was used to align the partial complex sequences. The maximum-likelihood (ML) bootstrap analysis was conducted using RAxML (Stamatakis 2006); bootstrap probability values were calculated from 1000 replicates. *Coffea canephora* (KU500324) and *Coffea arabica* (MK353212) were served as the out-group.

The complete *F. fragrans* plastid genome is a circular DNA molecule with the length of 154,416 bp, contains a large single copy region (LSC) of 84,405 bp and a small single copy region (SSC) of 18,285 bp, which were separated by a pair of inverted repeats (IR) regions of 25,863 bp. The overall GC content of the whole genome is 38.0%, and the corresponding values of the LSC, SSC, and IR regions are 36.0%, 31.8%, and 43.4%, respectively. The plastid genome contained 130 genes, including 85 protein-coding genes, 8 ribosomal RNA genes, and 37 transfer RNA genes. Phylogenetic analysis showed that *F. fragrans* clustered in a unique clade in Gentianaceae family (Figure 1). The determination of the complete plastid genome sequences provided new molecular data to illuminate the Gentianaceae family evolution.

**Figure 1.**
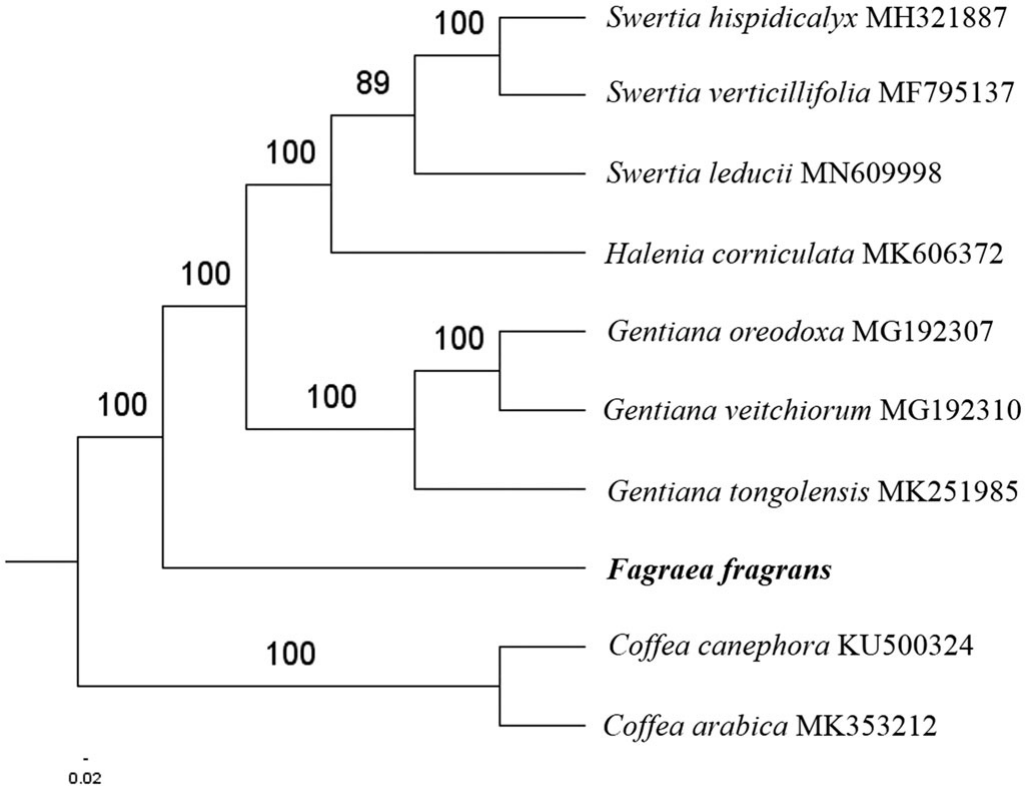
The maximum-likelihood tree based on the eight chloroplast genomes of Gentianaceae family. The bootstrap value based on 1000 replicates is shown on each node.

## References

[CIT0001] Jonville M, Baghdikian B, Ollivier E, Angenot L, Frederich M, Legault J. 2010. Anti-inflammatory potency of the traditionally used antimalarial plant *Fagraea fragrans*. Planta Med. 6(12):WSI–6.

[CIT0002] Jonville M-C, Capel M, Frédérich M, Angenot L, Dive G, Faure R, Azas N, Ollivier E, 2008. Fagraldehyde, a secoiridoid isolated from *Fagraea fragrans*. J Nat Prod. 71(12):2038–2040. 19053508 10.1021/np800291d

[CIT0003] Katoh K, Standley DM. 2013. MAFFT multiple sequence alignment software version 7: improvements in performance and usability. Mol Biol Evol. 30(4):772–780. 23329690 10.1093/molbev/mst010 PMC3603318

[CIT0004] Nguyen-Pouplin J, Tran H, Tran H, Phan TA, Dolecek C, Farrar J, Tran TH, Caron P, Bodo B, Grellier P. 2007. Antimalarial and cytotoxic activities of ethnopharmacologically selected medicinal plants from South Vietnam. J Ethnopharmacol. 109(3):417–427. 17010546 10.1016/j.jep.2006.08.011

[CIT0005] Pripdeevech P, Saansoomchai J. 2013. Antibacterial activity and chemical composition of essential oil and various extracts of *Fagraea fragrans* Roxb. flowers. Chiang Mai J Sci. 40(2):214–223.

[CIT0006] Stamatakis A. 2006. RAxML-VI-HPC: maximum likelihood-based phylogenetic analyses with thousands of taxa and mixed models. Bioinformatics. 22(21):2688–2690. 16928733 10.1093/bioinformatics/btl446

[CIT0007] Tillich M, Lehwark P, Pellizzer T, Ulbricht-Jones ES, Fischer A, Bock R, Greiner S. 2017. GeSeq-versatile and accurate annotation of organelle genomes. Nucleic Acids Res. 45(W1):W6–W11. 28486635 10.1093/nar/gkx391 PMC5570176

[CIT0008] Wong KM, Sugumaran M. 2012. Studies in Malesian Gentianaceae III: Cyrtophyllum reapplied to the *Fagraea fragrans* alliance. Gard Bull (Singapore). 64(2):497–510.

